# Supplementary material for: Iterative improvement in the automatic modular design of robot swarms
Source: PeerJ Comput Sci. 2020 Dec 7;6:e322. doi: 10.7717/peerj-cs.322 (PMC7924708; doi:10.7717/peerj-cs.322)
Supplement: Supplemental Information 3 [file peerj-cs-06-322-s003.zip › argos3/doc/api/standalone/a00468_source.html]

ARGoS: plugins/robots/foot-bot/simulator/footbot\_light\_rotzonly\_sensor.cpp Source File


- Main Page
- Related Pages
- Namespaces
- Classes
- Files

- File List
- File Members

# plugins/robots/foot-bot/simulator/footbot\_light\_rotzonly\_sensor.cpp

Go to the documentation of this file.

```
00001 
00007 #include <argos3/core/simulator/simulator.h>
00008 #include <argos3/core/simulator/entity/embodied_entity.h>
00009 #include <argos3/core/simulator/entity/composable_entity.h>
00010 #include <argos3/plugins/simulator/entities/light_entity.h>
00011 #include <argos3/plugins/simulator/entities/light_sensor_equipped_entity.h>
00012 
00013 #include "footbot_light_rotzonly_sensor.h"
00014 
00015 namespace argos {
00016 
00017    /****************************************/
00018    /****************************************/
00019 
00020    static CRange<Real> SENSOR_RANGE(0.0f, 1.0f);
00021    static CRadians SENSOR_SPACING      = CRadians(ARGOS_PI / 12.0f);
00022    static CRadians SENSOR_HALF_SPACING = SENSOR_SPACING * 0.5;
00023 
00024    /****************************************/
00025    /****************************************/
00026 
00027    static SInt32 Modulo(SInt32 n_value, SInt32 un_modulo) {
00028       while(n_value < 0) n_value += un_modulo;
00029       while(n_value >= un_modulo) n_value -= un_modulo;
00030       return n_value;
00031    }
00032 
00033    static Real ComputeReading(Real f_distance) {
00034       if(f_distance > 2.5f) {
00035          return 0.0f;
00036       }
00037       else {
00038          return ::exp(-f_distance * 2.0f);
00039       }
00040    }
00041 
00042    static Real ScaleReading(const CRadians& c_angular_distance) {
00043       if(c_angular_distance > CRadians::PI_OVER_TWO) {
00044          return 0.0f;
00045       }
00046       else {
00047          return (1.0f - 2.0f * c_angular_distance / CRadians::PI);
00048       }
00049    }
00050 
00051    /****************************************/
00052    /****************************************/
00053 
00054    CFootBotLightRotZOnlySensor::CFootBotLightRotZOnlySensor() :
00055       m_pcEmbodiedEntity(NULL),
00056       m_bShowRays(false),
00057       m_pcRNG(NULL),
00058       m_bAddNoise(false),
00059       m_cSpace(CSimulator::GetInstance().GetSpace()) {}
00060 
00061    /****************************************/
00062    /****************************************/
00063 
00064    void CFootBotLightRotZOnlySensor::SetRobot(CComposableEntity& c_entity) {
00065       try {
00066          m_pcEmbodiedEntity = &(c_entity.GetComponent<CEmbodiedEntity>("body"));
00067          m_pcControllableEntity = &(c_entity.GetComponent<CControllableEntity>("controller"));
00068          m_pcLightEntity = &(c_entity.GetComponent<CLightSensorEquippedEntity>("light_sensors"));
00069          m_pcLightEntity->Enable();
00070       }
00071       catch(CARGoSException& ex) {
00072          THROW_ARGOSEXCEPTION_NESTED("Can't set robot for the foot-bot light default sensor", ex);
00073       }
00074    }
00075 
00076    /****************************************/
00077    /****************************************/
00078 
00079    void CFootBotLightRotZOnlySensor::Init(TConfigurationNode& t_tree) {
00080       try {
00081          /* Show rays? */
00082          GetNodeAttributeOrDefault(t_tree, "show_rays", m_bShowRays, m_bShowRays);
00083          /* Parse noise level */
00084          Real fNoiseLevel = 0.0f;
00085          GetNodeAttributeOrDefault(t_tree, "noise_level", fNoiseLevel, fNoiseLevel);
00086          if(fNoiseLevel < 0.0f) {
00087             THROW_ARGOSEXCEPTION("Can't specify a negative value for the noise level of the light sensor");
00088          }
00089          else if(fNoiseLevel > 0.0f) {
00090             m_bAddNoise = true;
00091             m_cNoiseRange.Set(-fNoiseLevel, fNoiseLevel);
00092             m_pcRNG = CRandom::CreateRNG("argos");
00093          }
00094          m_tReadings.resize(m_pcLightEntity->GetNumSensors());
00095       }
00096       catch(CARGoSException& ex) {
00097          THROW_ARGOSEXCEPTION_NESTED("Initialization error in rot_z_only light sensor", ex);
00098       }
00099    }
00100 
00101    /****************************************/
00102    /****************************************/
00103    
00104    void CFootBotLightRotZOnlySensor::Update() {
00105       /* Erase readings */
00106       for(size_t i = 0; i < m_tReadings.size(); ++i) {
00107          m_tReadings[i].Value = 0.0f;
00108       }
00109       /* Get foot-bot orientation */
00110       CRadians cTmp1, cTmp2, cOrientationZ;
00111       m_pcEmbodiedEntity->GetOriginAnchor().Orientation.ToEulerAngles(cOrientationZ, cTmp1, cTmp2);
00112       /* Ray used for scanning the environment for obstacles */
00113       CRay3 cOcclusionCheckRay;
00114       cOcclusionCheckRay.SetStart(m_pcEmbodiedEntity->GetOriginAnchor().Position);
00115       CVector3 cRobotToLight;
00116       /* Buffer for the angle of the light wrt to the foot-bot */
00117       CRadians cAngleLightWrtFootbot;
00118       /* Buffers to contain data about the intersection */
00119       SEmbodiedEntityIntersectionItem sIntersection;
00120       /* List of light entities */
00121       CSpace::TMapPerType& mapLights = m_cSpace.GetEntitiesByType("light");
00122       /*
00123        * 1. go through the list of light entities in the scene
00124        * 2. check if a light is occluded
00125        * 3. if it isn't, distribute the reading across the sensors
00126        *    NOTE: the readings are additive
00127        * 4. go through the sensors and clamp their values
00128        */
00129       for(CSpace::TMapPerType::iterator it = mapLights.begin();
00130           it != mapLights.end();
00131           ++it) {
00132          /* Get a reference to the light */
00133          CLightEntity& cLight = *(any_cast<CLightEntity*>(it->second));
00134          /* Consider the light only if it has non zero intensity */
00135          if(cLight.GetIntensity() > 0.0f) {
00136             /* Set the ray end */
00137             cOcclusionCheckRay.SetEnd(cLight.GetPosition());
00138             /* Check occlusion between the foot-bot and the light */
00139             if(! GetClosestEmbodiedEntityIntersectedByRay(sIntersection,
00140                                                           cOcclusionCheckRay,
00141                                                           *m_pcEmbodiedEntity)) {
00142                /* The light is not occluded */
00143                if(m_bShowRays) {
00144                   m_pcControllableEntity->AddCheckedRay(false, cOcclusionCheckRay);
00145                }
00146                /* Get the distance between the light and the foot-bot */
00147                cOcclusionCheckRay.ToVector(cRobotToLight);
00148                /*
00149                 * Linearly scale the distance with the light intensity
00150                 * The greater the intensity, the smaller the distance
00151                 */
00152                cRobotToLight /= cLight.GetIntensity();
00153                /* Get the angle wrt to foot-bot rotation */
00154                cAngleLightWrtFootbot = cRobotToLight.GetZAngle();
00155                cAngleLightWrtFootbot -= cOrientationZ;
00156                /*
00157                 * Find closest sensor index to point at which ray hits footbot body
00158                 * Rotate whole body by half a sensor spacing (corresponding to placement of first sensor)
00159                 * Division says how many sensor spacings there are between first sensor and point at which ray hits footbot body
00160                 * Increase magnitude of result of division to ensure correct rounding
00161                 */
00162                Real fIdx = (cAngleLightWrtFootbot - SENSOR_HALF_SPACING) / SENSOR_SPACING;
00163                SInt32 nReadingIdx = (fIdx > 0) ? fIdx + 0.5f : fIdx - 0.5f;
00164                /* Set the actual readings */
00165                Real fReading = cRobotToLight.Length();
00166                /*
00167                 * Take 6 readings before closest sensor and 6 readings after - thus we
00168                 * process sensors that are with 180 degrees of intersection of light
00169                 * ray with robot body
00170                 */
00171                for(SInt32 nIndexOffset = -6; nIndexOffset < 7; ++nIndexOffset) {
00172                   UInt32 unIdx = Modulo(nReadingIdx + nIndexOffset, 24);
00173                   CRadians cAngularDistanceFromOptimalLightReceptionPoint = Abs((cAngleLightWrtFootbot - m_tReadings[unIdx].Angle).SignedNormalize());
00174                   /*
00175                    * ComputeReading gives value as if sensor was perfectly in line with
00176                    * light ray. We then linearly decrease actual reading from 1 (dist
00177                    * 0) to 0 (dist PI/2)
00178                    */
00179                   m_tReadings[unIdx].Value += ComputeReading(fReading) * ScaleReading(cAngularDistanceFromOptimalLightReceptionPoint);
00180                }
00181             }
00182             else {
00183                /* The ray is occluded */
00184                if(m_bShowRays) {
00185                   m_pcControllableEntity->AddCheckedRay(true, cOcclusionCheckRay);
00186                   m_pcControllableEntity->AddIntersectionPoint(cOcclusionCheckRay, sIntersection.TOnRay);
00187                }
00188             }
00189          }
00190       }
00191       /* Apply noise to the sensors */
00192       if(m_bAddNoise) {
00193          for(size_t i = 0; i < 24; ++i) {
00194             m_tReadings[i].Value += m_pcRNG->Uniform(m_cNoiseRange);
00195          }
00196       }
00197       /* Trunc the reading between 0 and 1 */
00198       for(size_t i = 0; i < 24; ++i) {
00199          SENSOR_RANGE.TruncValue(m_tReadings[i].Value);
00200       }
00201    }
00202 
00203    /****************************************/
00204    /****************************************/
00205 
00206    void CFootBotLightRotZOnlySensor::Reset() {
00207       for(UInt32 i = 0; i < GetReadings().size(); ++i) {
00208          m_tReadings[i].Value = 0.0f;
00209       }
00210    }
00211 
00212    /****************************************/
00213    /****************************************/
00214 
00215    REGISTER_SENSOR(CFootBotLightRotZOnlySensor,
00216                    "footbot_light", "rot_z_only",
00217                    "Carlo Pinciroli [ilpincy@gmail.com]",
00218                    "1.0",
00219                    "The foot-bot light sensor (optimized for 2D).",
00220                    "This sensor accesses a set of light sensors. The sensors all return a value\n"
00221                    "between 0 and 1, where 0 means nothing within range and 1 means the perceived\n"
00222                    "light saturates the sensor. Values between 0 and 1 depend on the distance of\n"
00223                    "the perceived light. Each reading R is calculated with R=(I/x)^2, where x is the\n"
00224                    "distance between a sensor and the light, and I is the reference intensity of the\n"
00225                    "perceived light. The reference intensity corresponds to the minimum distance at\n"
00226                    "which the light saturates a sensor. The reference intensity depends on the\n"
00227                    "individual light, and it is set with the \"intensity\" attribute of the light\n"
00228                    "entity. In case multiple lights are present in the environment, each sensor\n"
00229                    "reading is calculated as the sum of the individual readings due to each light.\n"
00230                    "In other words, light wave interference is not taken into account. In\n"
00231                    "controllers, you must include the ci_light_sensor.h header.\n\n"
00232                    "REQUIRED XML CONFIGURATION\n\n"
00233                    "  <controllers>\n"
00234                    "    ...\n"
00235                    "    <my_controller ...>\n"
00236                    "      ...\n"
00237                    "      <sensors>\n"
00238                    "        ...\n"
00239                    "        <footbot_light implementation=\"rot_z_only\" />\n"
00240                    "        ...\n"
00241                    "      </sensors>\n"
00242                    "      ...\n"
00243                    "    </my_controller>\n"
00244                    "    ...\n"
00245                    "  </controllers>\n\n"
00246                    "OPTIONAL XML CONFIGURATION\n\n"
00247                    "It is possible to draw the rays shot by the light sensor in the OpenGL\n"
00248                    "visualization. This can be useful for sensor debugging but also to understand\n"
00249                    "what's wrong in your controller. In OpenGL, the rays are drawn in cyan when\n"
00250                    "they are not obstructed and in purple when they are. In case a ray is\n"
00251                    "obstructed, a black dot is drawn where the intersection occurred.\n"
00252                    "To turn this functionality on, add the attribute \"show_rays\" as in this\n"
00253                    "example:\n\n"
00254                    "  <controllers>\n"
00255                    "    ...\n"
00256                    "    <my_controller ...>\n"
00257                    "      ...\n"
00258                    "      <sensors>\n"
00259                    "        ...\n"
00260                    "        <footbot_light implementation=\"rot_z_only\"\n"
00261                    "                       show_rays=\"true\" />\n"
00262                    "        ...\n"
00263                    "      </sensors>\n"
00264                    "      ...\n"
00265                    "    </my_controller>\n"
00266                    "    ...\n"
00267                    "  </controllers>\n\n"
00268                    "It is possible to add uniform noise to the sensors, thus matching the\n"
00269                    "characteristics of a real robot better. This can be done with the attribute\n"
00270                    "\"noise_level\", whose allowed range is in [-1,1] and is added to the calculated\n"
00271                    "reading. The final sensor reading is always normalized in the [0-1] range.\n\n"
00272                    "  <controllers>\n"
00273                    "    ...\n"
00274                    "    <my_controller ...>\n"
00275                    "      ...\n"
00276                    "      <sensors>\n"
00277                    "        ...\n"
00278                    "        <footbot_light implementation=\"rot_z_only\"\n"
00279                    "                       noise_level=\"0.1\" />\n"
00280                    "        ...\n"
00281                    "      </sensors>\n"
00282                    "      ...\n"
00283                    "    </my_controller>\n"
00284                    "    ...\n"
00285                    "  </controllers>\n\n"
00286                    "OPTIONAL XML CONFIGURATION\n\n"
00287                    "None.\n",
00288                    "Usable"
00289       );
00290 
00291 }
```

---

Generated on 10 Jul 2018 for ARGoS by 
 1.6.1
